# Common methods for fecal sample storage in field studies yield consistent signatures of individual identity in microbiome sequencing data

**DOI:** 10.1038/srep31519

**Published:** 2016-08-16

**Authors:** Ran Blekhman, Karen Tang, Elizabeth A. Archie, Luis B. Barreiro, Zachary P. Johnson, Mark E. Wilson, Jordan Kohn, Michael L. Yuan, Laurence Gesquiere, Laura E. Grieneisen, Jenny Tung

**Affiliations:** 1 Department of Genetics, Cell Biology, and Development, University of Minnesota, Minneapolis, MN 55108, USA; 2 Department of Ecology, Evolution, and Behavior, University of Minnesota, Minneapolis, MN 55108, USA; 3 Department of Biological Sciences, University of Notre Dame, Notre Dame, IN 45665, USA; 4 Institute of Primate Research, National Museums of Kenya, Nairobi 00502, Kenya; 5 Department of Pediatrics, Sainte-Justine Hospital Research Centre, University of Montreal, Montreal, Quebec, H3T 1C5 Canada; 6 Yerkes National Primate Research Center, Emory University, Atlanta, GA 30322, USA; 7 Department of Evolutionary Anthropology, Duke University, Durham, NC 27708, USA; 8 Department of Biology, Duke University, Durham, NC 27708, USA; 9 Duke Population Research Institute, Duke University, Durham NC 27708, USA

## Abstract

Field studies of wild vertebrates are frequently associated with extensive collections of banked fecal samples—unique resources for understanding ecological, behavioral, and phylogenetic effects on the gut microbiome. However, we do not understand whether sample storage methods confound the ability to investigate interindividual variation in gut microbiome profiles. Here, we extend previous work on storage methods for gut microbiome samples by comparing immediate freezing, the gold standard of preservation, to three methods commonly used in vertebrate field studies: lyophilization, storage in ethanol, and storage in RNAlater. We found that the signature of individual identity consistently outweighed storage effects: alpha diversity and beta diversity measures were significantly correlated across methods, and while samples often clustered by donor, they never clustered by storage method. Provided that all analyzed samples are stored the same way, banked fecal samples therefore appear highly suitable for investigating variation in gut microbiota. Our results open the door to a much-expanded perspective on variation in the gut microbiome across species and ecological contexts.

Noninvasive collection is often the only feasible approach for obtaining samples from wild vertebrates, especially in threatened or endangered species^1^. Fecal samples are especially common, as they can be collected without disrupting study subjects, can often be unambiguously assigned to donors, and can be longitudinally collected from the same animal over time. Such samples also contain abundant information about the genetics, endocrinology, and parasite burden of the animals from which they are obtained. For these reasons, fecal samples may be the most extensively banked sample type available for wild vertebrates.

Such collections represent potentially invaluable resources for understanding interindividual, temporal, and spatial variation in the gut microbiome in comparative or conservation contexts. However, sample storage methods vary widely across studies, and in most cases, samples were not collected with microbiome analyses in mind. To assess the potential for mining existing sample banks, we investigated how three common field storage methods—storage in ethanol, storage in RNAlater, or storage in ethanol followed by lyophilization (typical for studies of steroid hormone levels)—affect gut microbiome diversity and composition estimates, compared to the gold standard of immediate freezing. We were particularly interested in comparing the roles of storage method versus individual identity, as the latter captures the biological variation generally of most interest to researchers (whereas storage-driven variance is purely technical). Although storage methods often explain substantial variation in microbiome composition when all other sources of variance are controlled^2^,^3^, the degree to which they confound other analyses depends on their importance *relative* to the effects of biologically interesting variation (interindividual, temporal, and environmental). With one recent exception focused on humans and domestic dogs^4^, previous studies have focused on small numbers of study subjects (n ≤ 5), limiting their ability to evaluate this question^2^,^3^,^5^,^6^,^7^,^8^,^9^,^10^.

Here, we compared fecal samples collected from 13 captive adult rhesus macaques (*Macaca mulatta*), with each sample divided into four aliquots (one per storage condition we studied). We then evaluated individual identity versus technical storage effects on both alpha diversity and beta diversity estimates from high sequencing-depth 16S rRNA profiles. Notably, this study design provides a conservative estimate of the contribution of individual identity relative to technical variance, as all animals were sampled in standardized housing, dietary, and social group conditions.

## Results

For each individual in the study, we collected a fresh stool sample and divided it into four aliquots (n = 52 samples; Table S1). These were stored for 2–6 days prior to DNA extraction via: 1) immediate freezing at −20 °C; 2) immersion in absolute ethanol; 3) immersion in the preservative RNAlater; or 4) immersion in ethanol followed by lyophilization to powder (used for steroid hormone analysis and sometimes for genetic samples^11^,^12^; we subsequently refer to this condition simply as “lyophilized”). For each sample, we then generated amplicon libraries targeting the bacterial 16S rRNA V4 region^13^, multiplexed them for sequencing, and performed quality filtering and OTU abundance estimation in QIIME^14^ (see Methods). We eliminated one ethanol sample because it generated very few reads; all remaining samples were rarefied to 54,633 reads for subsequent analyses. We identified 21,006 OTUs overall (mean per sample = 1,656 ± 237 s.d.; Table S1).

**Figure 1:**
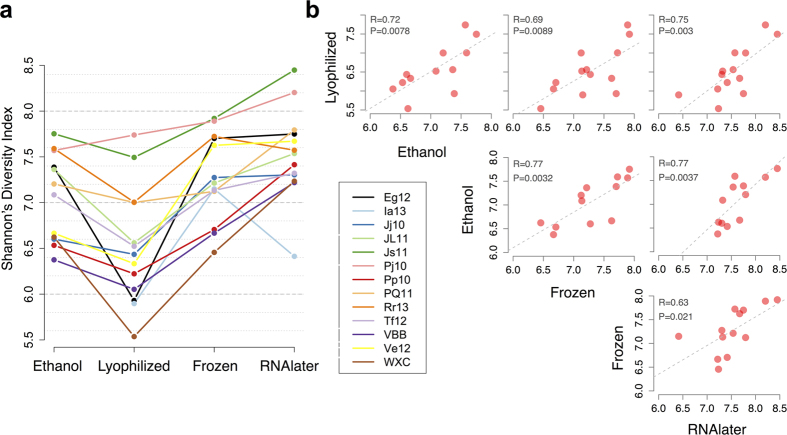
Storage effects on alpha diversity. (**a**) Shannon’s Diversity Index (SDI) values (y-axis) shown as a function of storage method (x-axis), with each individual plotted in a different color. Lyophilized samples have lower SDI values than other storage methods (Tukey’s HSD, lyophilized-frozen: p = 0.004; lyophilized-RNAlater: p = 7.6 × 10^−5^; lyophilized-ethanol: p = 0.063; Table S2). (**b**) SDI values are significantly correlated within individuals, between all storage methods (Pearson’s correlation, p < 0.05). Each dot represents an individual, and each panel shows the correlation between SDI values obtained from two different storage methods.

The resulting data recapitulated previous observations showing storage condition effects on mean alpha diversity^2^,^3^,^5^,^7^. In our case, lyophilized samples exhibited lower Shannon’s Diversity Index (SDI) values relative to other conditions (Tukey’s HSD: p between 7.6 × 10^−5^ and 0.063), and samples stored in RNAlater exhibited somewhat higher values, although this comparison was only significant in comparison to the lyophilized condition (p = 7.6 × 10^−5^; Fig. 1a; Table S2). Among the OTUs that were present at an abundance ≥0.1% in any sample (n = 696 OTUs), none were identified as present in one condition but absent in the other three storage conditions, and only 11—primarily of the order Clostridiales—were absent in one storage condition but present in the others (always missing in the lyophilized condition: Table S3). Of these 11 OTUs, 8 were very low abundance (<0.05% relative abundance) in all but one female.

Overall, SDI values retained a strong signature of individual identity. Specifically, SDI values were significantly correlated across samples from the same individual across storage methods (Fig. 1b). Further, although we observed several changes in the rank order of SDI values by individual, across conditions (Fig. 1a), individual identity explained a larger proportion of variance in SDI across samples than storage condition (ANOVA: 50% versus 36%). We obtained qualitatively similar but weaker results for the number of OTUs identified in each sample (Figure S1), suggesting that the combination of species richness and evenness captured by SDI is more stable than richness alone.

**Figure 2:**
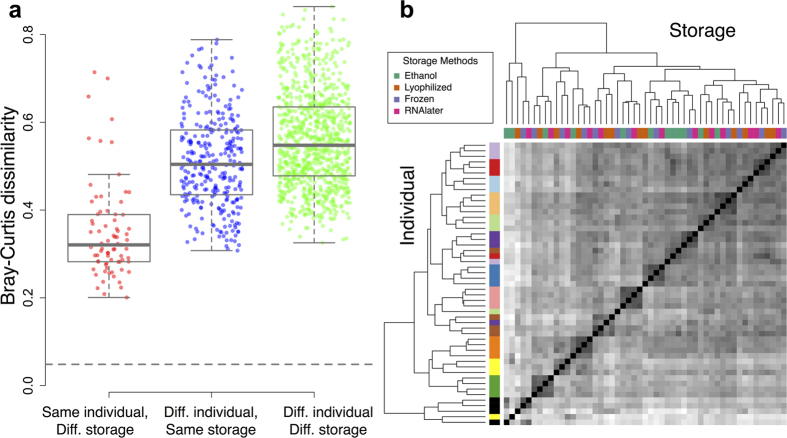
Storage effects on beta diversity. **(a)** Bray-Curtis dissimilarity values (y-axis) comparing the same individual from samples collected under different storage conditions (red), different individuals with samples collected under the same storage conditions (blue), and different individuals with samples collected under different storage conditions (green). Median Bray-Curtis dissimilarity calculated from subsampling reads from the same sample (i.e., the minimum dissimilarity due to read resampling alone) is indicated by the gray dashed line. Because of the large number of data points, all pairwise comparisons are highly significant (Wilcoxon Rank Sum test, p < 1 × 10^−9^). However, the dissimilarity values for same individual/different storage are much lower on average (mean = 0.35 ± 0.11 s.d.) than dissimilarity values measured between individuals in either the same (mean = 0.51 ± 0.11 s.d.) or different (0.56 ± 0.11 s.d.) storage conditions. **(b)** Bray-Curtis dissimilarities cluster more strongly by individual (colors along the left-hand sidebar, with one color per individual) than by storage method (colors shown on the top, next to the dendrogram, and in the boxed legend).

Beta diversity measures of community similarity (Tables S4–S6) were also more influenced by individual identity than sample storage condition. 66.3% of variation in taxonomic abundance could be explained by individual identity, compared to 14.3% by storage method (PERMANOVA on a Bray-Curtis dissimilarity matrix; p < 0.001 for both predictors; similar results were obtained for weighted UniFrac: 53.1% versus 26.2%; and for unweighted UniFrac: 43.2% versus 8.2%). Bray-Curtis dissimilarities were much higher for pairs of samples collected from different individuals (mean = 0.51 ± 0.11 s.d. within condition; 0.56 ± 0.11 s.d. between conditions) than for samples collected from the same individual using different storage conditions (mean = 0.35 ± 0.11 s.d., Fig. 2a; see also Figure S2). Samples from the same individual, but not storage condition, also clustered together in a hierarchical clustering analysis using either Bray-Curtis or unweighted UniFrac measures (Fig. 2b; Figure S3a; no clustering, either by individual or storage condition, was observable using weighted UniFrac: Figure S3b). Most importantly, relative distances between individuals remained consistent across storage conditions. For example, pairwise correlations between Bray-Curtis dissimilarity matrices calculated separately for each condition were highly correlated (r = 0.59 to 0.88, all p < 0.005; Table S7), with similar patterns observed using weighted or unweighted UniFrac (Tables S8 and S9).

## Discussion

Together, our results indicate that, while mean alpha and beta diversity values are sometimes altered by storage condition, biologically relevant signatures of individual identity tend to be retained, especially for measures of beta diversity. Our findings agree with previous studies using fewer individuals^6^,^7^,^8^,^10^, and extend them to three of the most commonly used storage methods in vertebrate field studies. For many types of studies, storage condition *per se* may therefore be less important than maintaining consistency in storage methods within a data set (suggesting caution when performing comparisons across data sets that use different methods, unless the variable of interest has a considerably larger effect size than storage condition). Notably, in natural populations, interindividual differences in diet, social contacts, and habitat will likely amplify the signature of individual identity even further.

Thus, while immediate freezing will always be preferred when possible, when it is not possible—as is often the case in field studies—other methods provide reasonable alternatives. Although the differences are small, alpha diversity tended to be more highly correlated between frozen and ethanol-stored samples than frozen and RNAlater-stored samples, suggesting that ethanol may be as good or better of a storage medium than more expensive options (consistent with previous work^3^,^4^,^15^,^16^).

Together, our findings support the utility of using banked fecal sample collections from field studies for analyses of gut microbiome variation. These collections are not only substantial (ranging up to tens of thousands of samples), but are also often longitudinal, complemented by extensive demographic and behavioral metadata, and focused on species of particular conservation concern. As a note of caution, our study examined only short-term storage, so the long-term effects of storage in alternative media remain important to examine (but see^4^, where storage up to 8 weeks produced minimal sample preservation effects). However, given appropriate controls for sample age and DNA quality, our results suggest that sample banks from field studies may represent extraordinary, largely untapped resources for understanding the causes, consequences, and diversity of gut microbial structure.

## Methods

### Study subjects, sample collection, and sequencing

Study subjects were 13 adult female rhesus macaques (*Macaca mulatta*), members of 7 different social groups housed at the Yerkes National Primate Research Center (YNPRC). These groups were formed as part of a separate study on the relationship between dominance rank and gene regulation. All groups were maintained in standardized indoor-outdoor housing runs (25 m × 25 m per run), under standardized demographic (5 adult females per group), dietary, and observational conditions.

Fecal samples were collected within 10–15 minutes after deposition and subdivided into four equal subsamples. The first subsample was frozen immediately at −20 °C; the second subsample immersed in the commercial preservative RNAlater (Life Technologies, Carlsbad, CA); and the third and fourth subsamples immersed in absolute ethanol. After one day of room temperature storage, one of the ethanol-stored subsamples was lyophilized following standard methods used to process fecal samples for steroid hormone analysis in primate field studies (ethanol evaporation followed by 0.1 millibar of vacuum pressure at −50 °C^11^). DNA from all samples was extracted using MO BIO’s PowerSoil DNA Isolation kit (MO BIO Laboratories, Inc., Carlsbad, CA). For lyophilized samples, extractions were obtained from 0.05 g of sample instead of 0.25 g to avoid complete absorption of liquid in the first steps of the DNA extraction. We note that the lyophilization procedure involves additional sample handling relative to the other conditions we tested, which could introduce additional opportunity for sample contamination. However, the general agreement between lyophilized samples and the other samples suggests this is not a major concern if proper sterile technique is employed.

16S rRNA library preparation (targeting the V4 region) and sequencing were conducted at the University of Minnesota Genomics Center, using forward and reverse primers 515F and 806R^13^ followed by amplification with indexing primers and 300 bp paired-end sequencing on a MiSeq flowcell (see Table S1 for sample-specific details).

All animal work was carried out in accordance with the relevant guidelines and regulations established by AALAC International (YNPRC is a fully accredited animal housing facility). This research was conducted under Institutional Animal Care and Use Committee protocols approved by Emory University (IACUC YER-2001677-040715GA) and Duke University (IACUC A079-12-03).

### Data analysis

Following sample demultiplexing, primer sequences were removed from the raw reads using CutAdapt v.1.7.1^17^. Because CutAdapt does not always detect reverse primers effectively, the first 29 base pairs (theoretically the primer and linker sequences) were removed from reverse reads. Reads were truncated at the first base pair with a PHRED quality score ≤3, and forward and reverse reads were then merged using USEARCH v6.1^18^. Read pairs that failed to merge were discarded. We used QIIME v1.8 to conduct further quality control filtering^14^, using default parameters except for the minimum acceptable per-base Phred score parameter, which we increased from 4 to 20. Putative chimeric sequences were identified using UCHIME (implemented in USEARCH v6.1^19^), and sequences were discarded from the sample when both reference-based (against the RDP Gold training database v9^20^) and *de novo* abundance-based methods flagged them as likely chimeras.

To identify operational taxonomic units (OTUs) in our data set, we used the open-reference OTU picking pipeline in QIIME. Specifically, the set of chimera-filtered reads was first clustered using the UCLUST v1.2.22 algorithm and the GreenGenes database (May 2013 release^21^), with a 97% identity threshold. Sequences that failed to cluster against the reference database were then clustered *de novo*, with sequences that failed both clustering attempts discarded. A representative sequence for each cluster was selected based on the most abundant sequence, and then aligned using PyNAST v1.2.2^22^. Sequences that failed to align were discarded. Taxonomic identity was assigned to aligned OTUs using the RDP classifier v2.2^23^, retrained to the May 2013 release of the GreenGenes database^21^. Singleton OTUs were removed from the OTU table as they tend to be enriched for sequencing errors. We also removed one sample due to low read count.

For all subsequent analyses, we rarefied the OTU table to 54,633 reads per sample using the QIIME v 1.8.0 script single_rarefaction.py. Subsampling reads from individuals with uniformly high coverage across storage conditions supported the stability of our summary statistics at this level of rarefaction (Figure S4). We calculated alpha diversity measures using the QIIME v 1.8.0 script alpha_diversity.py, and beta diversity measures using the corresponding QIIME v 1.8.0 script beta_diversity.py^14^. To estimate the minimum beta diversity dissimilarity due to random resampling error, we used high sequencing depth samples for which at least five times the number of rarefied reads were available (273,165 reads; n = 12). We drew five random subsamples from the total quality-filtered read count for each of these samples. We then calculated all pairwise Bray-Curtis dissimilarity values between subsamples from the same original sample. The median of these dissimilarity values is shown as the dashed line in Fig. 2a (and for weighted and unweighted UniFrac analyses in Figure S2).

All statistical analyses on alpha and beta diversity values were conducted in R v 3.1.1^24^ using either the R base packages or, for PERMANOVA, the R package *vegan*^25^ and the R package *ade4* v 1.7–2^26^.

### Data Availability

All raw read data and associated sample metadata are available in the NCBI Short Read Archive (SRP072517).

## Additional Information

**How to cite this article**: Blekhman, R. *et al*. Common methods for fecal sample storage in field studies yield consistent signatures of individual identity in microbiome sequencing data. *Sci. Rep.* **6**, 31519; doi: 10.1038/srep31519 (2016).

## Supplementary Material

Supplementary Information

Supplementary Information
