# Supplementary material for: Common methods for fecal sample storage in field studies yield consistent signatures of individual identity in microbiome sequencing data
Source: Sci Rep. 2016 Aug 16;6:31519. doi: 10.1038/srep31519 (PMC4985740; doi:10.1038/srep31519)
Supplement: Supplementary Information [file srep31519-s1.pdf]

Supplementary Materials for: Common methods for fecal sample storage in field studies yield consistent signatures of individual identity in microbiome sequencing data

Authors: Ran Blekhman[1,2], Karen Tang[1,2], Elizabeth A. Archie[3,4], Luis B. Barreiro[5], Zachary P. Johnson[6], Mark E. Wilson[6], Jordan Kohn[6], Michael L. Yuan[7], Laurence Gesquiere[8], Laura E. Grieneisen[3], Jenny Tung[4,7,8,9]

[1]Department of Genetics, Cell Biology, and Development, University of Minnesota, Minneapolis, MN 55108, USA

[2]Department of Ecology, Evolution, and Behavior, University of Minnesota, Minneapolis, MN 5510, USA.

[3]Department of Biological Sciences, University of Notre Dame, Notre Dame, IN 45665, USA

[4]Institute of Primate Research, National Museums of Kenya, Nairobi 00502, Kenya

[5]Department of Pediatrics, Sainte-Justine Hospital Research Centre, University of Montreal, Montreal, Quebec, Canada. H3T 1C5

[6]Yerkes National Primate Research Center, Emory University, Atlanta, GA 30322, USA

[7]Department of Evolutionary Anthropology, Duke University, Durham, NC 27708, USA

[8]Department of Biology, Duke University, Durham, NC 27708, USA

[9]Duke Population Research Institute, Duke University, Durham NC 27708, USA

Supplementary Material Contents

1. Supplementary Tables

Supplementary Tables are provided as a single .xls file, with the following spreadsheets:

Table S1. Sample information and summary of sequencing results
Table S2. Differences in mean SDI across storage conditions
Table S3. Taxa consistently absent in the lyophilized condition
Table S4. Bray-Curtis dissimilarity values between all samples
Table S5. Unweighted UniFrac distances between all samples
Table S6. Weighted UniFrac distances between all samples
Table S7. Correlations between Bray-Curtis dissimilarity matrices obtained from different storage conditions
Table S8. Correlations between Weighted UniFrac dissimilarity matrices obtained from different storage conditions
Table S9. Correlations between Unweighted UniFrac dissimilarity matrices obtained from different storage conditions.

2. Supplementary Figures

Figure S1. Pairwise correlations for species richness across storage conditions
Figure S2. Weighted and unweighted UniFrac dissimilarity values between different sample types
Figure S3. Hierarchical clustering plots based on unweighted and weighted Unifrac dissimilarity measures
Figure S4. Statistical robustness to rarefaction

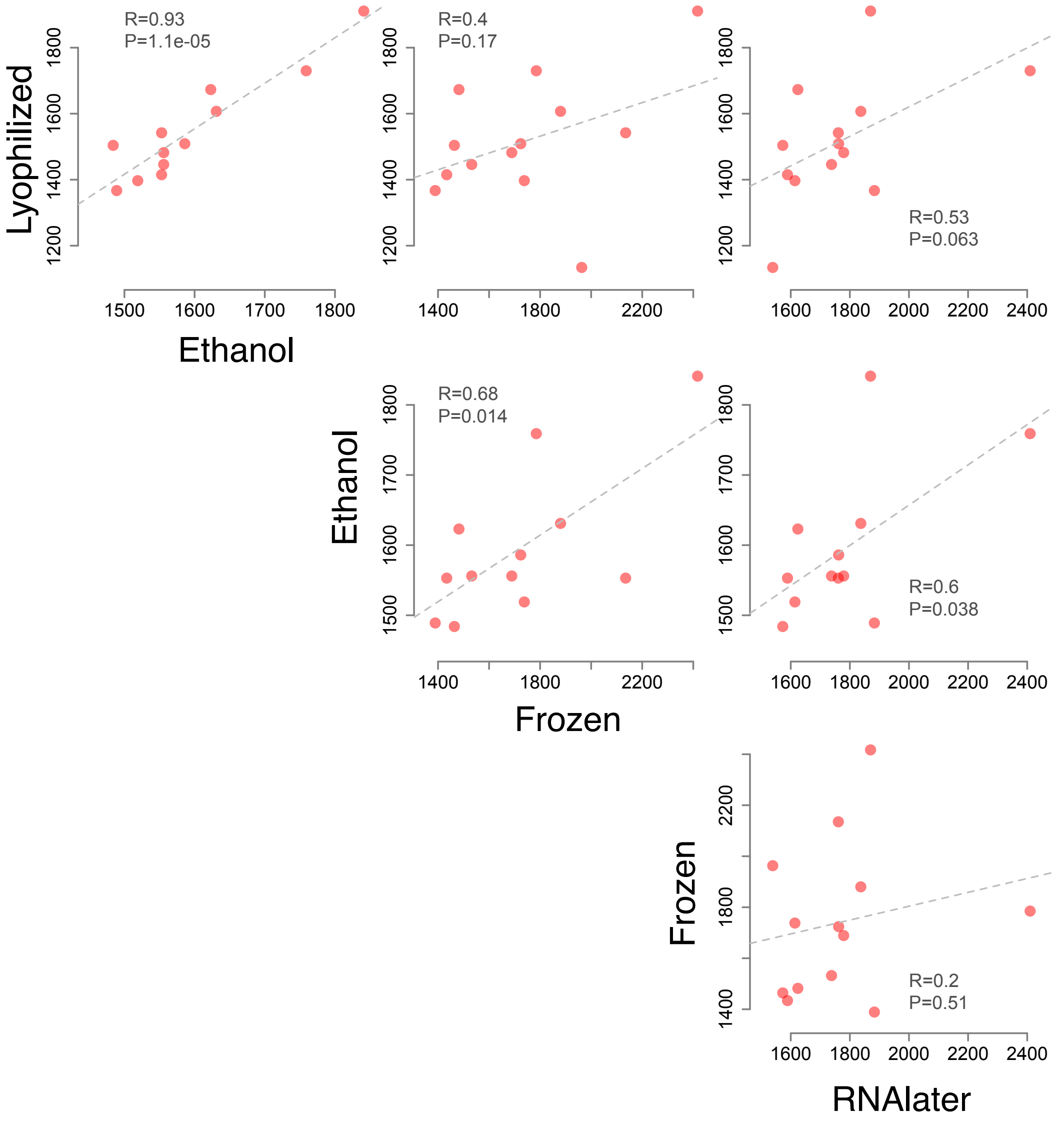

Figure S1. Pairwise correlations for species richness across storage conditions. Correlation within individual, between storage methods, for the number of OTUs detected in each sample (Pearson's correlation). Each dot represents an individual, and each panel shows the correlation between values in two different storage methods.

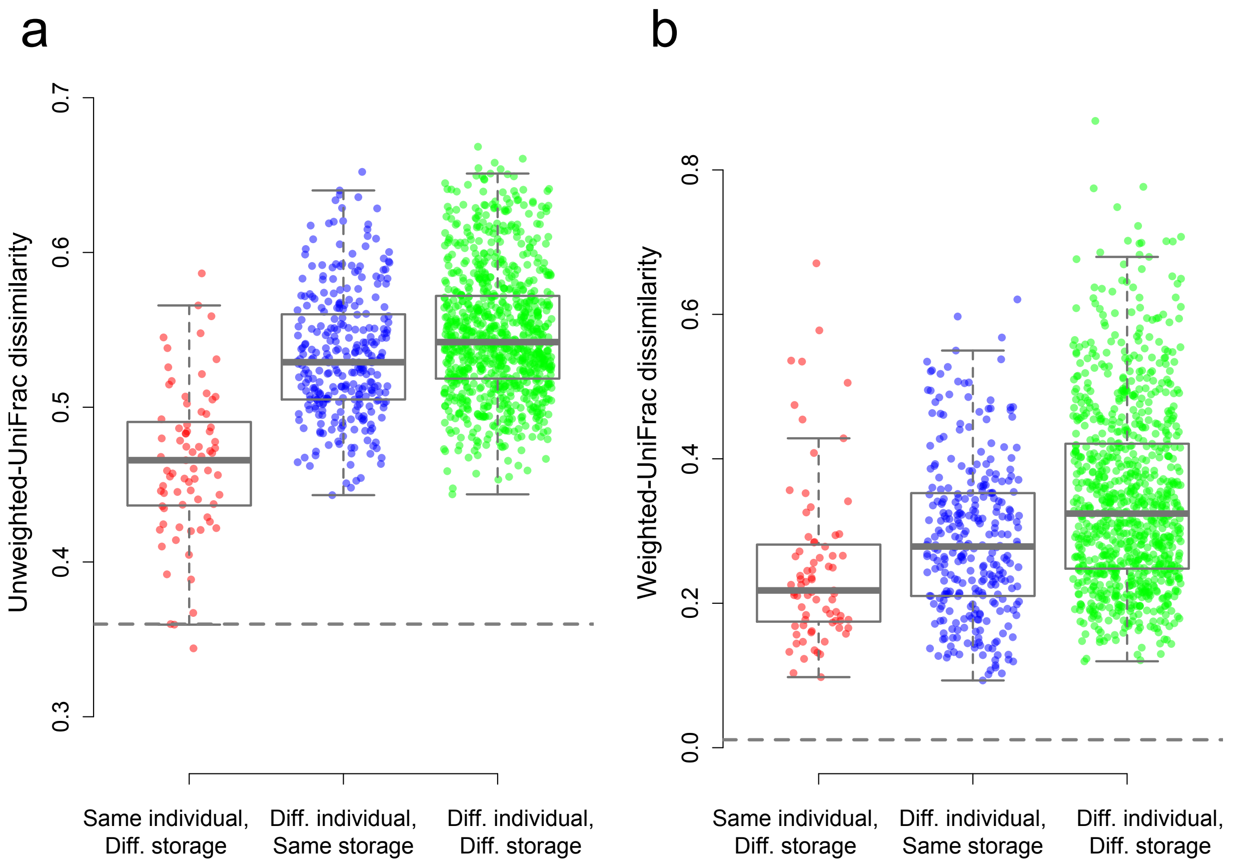

Figure S2. Weighted and unweighted UniFrac dissimilarity values between different sample types. (A) Weighted UniFrac dissimilarities comparing the same individual from samples collected under different storage conditions (red), different individuals with samples collected under the same storage conditions (blue), and different individuals with samples collected under different storage conditions (green). (B) As in A, but for unweighted UniFrac dissimilarities. In both A and B, the grey dashed line indicates the median dissimilarity value calculated from subsampling reads from the same sample (i.e., the minimum dissimilarity due to read resampling alone).

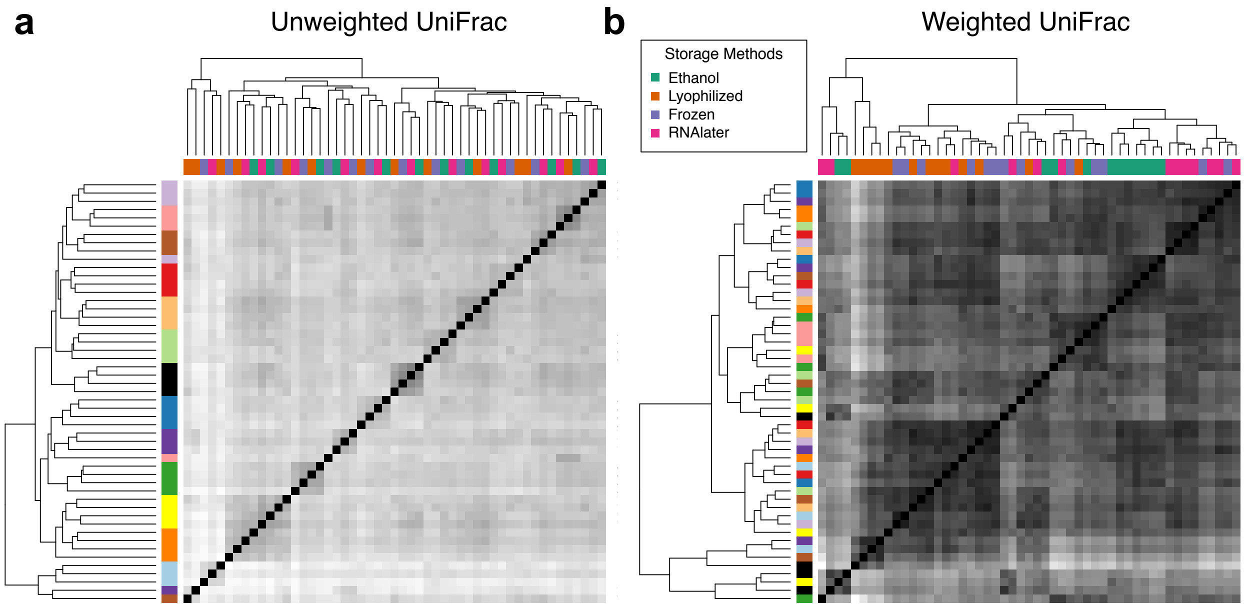

Figure S3. Hierarchical clustering plots based on unweighted and weighted UniFrac dissimilarity measures. Like Bray-Curtis dissimilarities (main text, Figure 2), (A) unweighted UniFrac dissimilarities cluster more strongly by individual (colors along the lefthand sidebar, with one color per individual) than by storage method (colors shown on the top). (B) Weighted UniFrac similarities show no clear clustering by individual or storage method.

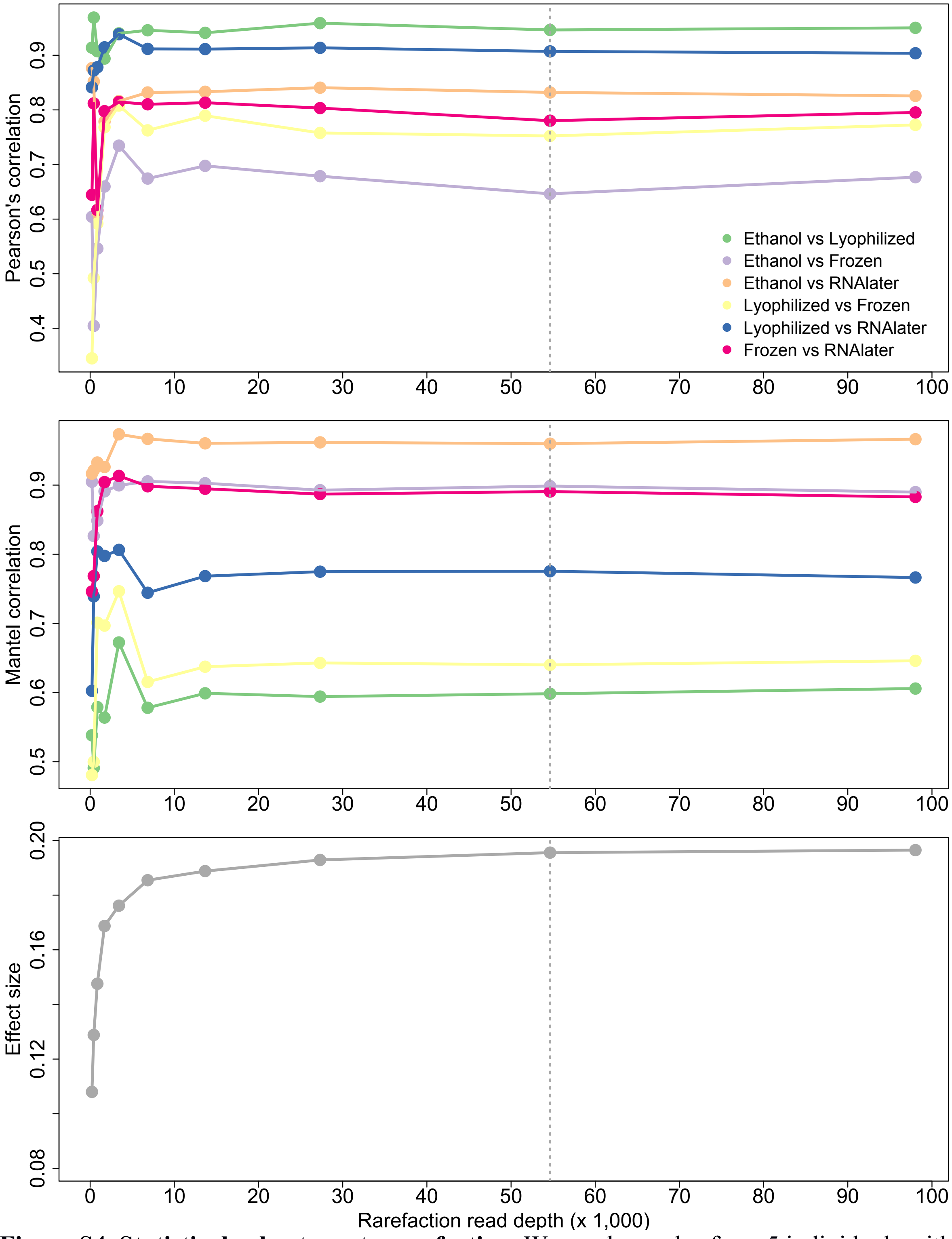

Figure S4. Statistical robustness to rarefaction. We used samples from 5 individuals with high coverage across all four storage conditions to test whether the rarefied sequencing coverage used in our main analysis (gray dashed line) was sufficient to produce stable estimates of (i) SDI correlations across storage condition (top panel); (ii) Mantel test correlations of Bray-Curtis dissimilarity values across storage conditions (middle panel); and (iii) Wilcoxon test statistics for

the difference in mean pairwise Bray-Curtis dissimilarity values when comparing samples from the same individual stored in different conditions to samples from different individuals stored in the same condition (bottom panel). In all three cases, test statistics stabilize by a depth of 20,000-30,000 rarefied reads, lower than the read depth used in our actual analyses.
